# Antibiogram of Medical Intensive Care Unit at Tertiary Care Hospital Setting of Pakistan

**DOI:** 10.7759/cureus.809

**Published:** 2016-09-29

**Authors:** Aayesha Qadeer, Aftab Akhtar, Qurat Ul Ain, Shoab Saadat, Salman Mansoor, Salman Assad, Wasib Ishtiaq, Abid Ilyas, Ali Y Khan, Yousaf Ajam

**Affiliations:** 1 Department of Pulmonology & Critical Care Medicine, Shifa International Hospital, Islamabad, Pakistan; 2 Medical Officer, Shifa International Hospital, Islamabad, Pakistan; 3 Nephrology, Shifa International Hospital, Islamabad, Pakistan; 4 Department of Neurology, Shifa International Hospital, Islamabad, Pakistan; 5 Department of Neurology & Neurosurgery, Shifa Tameer-e-Millat University, Islamabad, Pakistan; 6 Department of Internal Medicine & Infectious Diseases, Shifa International Hospital, Islamabad, Pakistan; 7 Shifa International Hospital, Islamabad, Pakistan

**Keywords:** resistance, bacteria, antibiogram

## Abstract

Objective: To determine the frequency of micro-organisms causing sepsis as well as to determine the antibiotic susceptibility and resistance of microorganisms isolated in a medical intensive care unit.

Materials and methods: This is a cross-sectional analysis of 802 patients from a medical intensive care unit (ICU) of Shifa International Hospital, Islamabad, Pakistan over a one-year period from August 2015 to August 2016. Specimens collected were from blood, urine, endotracheal secretions, catheter tips, tissue, pus swabs, cerebrospinal fluid, ascites, bronchoalveolar lavage (BAL), and pleural fluid. All bacteria were identified by standard microbiological methods, and antibiotic sensitivity/resistance was performed using the disk diffusion technique, according to Clinical and Laboratory Standards Institute (CLSI) guidelines. Data was collected using a critical care unit electronic database and data analysis was done by using  the Statistical Package for Social Sciences (SPSS), version 20 (IBM SPSS Statistics, Armonk, NY).

Results: Gram-negative bacteria were more frequent as compared to gram-positive bacteria. Most common bacterial isolates were Acinetobacter (15.3%), Escherichia coli (15.3%), Pseudomonas aeruginosa (13%), and Klebsiella pneumoniae (10.2%), whereas Enterococcus (7%) and methicillin-resistant staphylococcus aureus (MRSA) (6.2%) were the two most common gram-positive bacteria. For Acinetobacter, colistin was the most effective antibiotic (3% resistance). For E.coli, colistin (0%), tigecycline (0%), amikacin (7%), and carbapenems (10%) showed low resistance. Pseudomonas aeruginosa showed low resistance to colistin (7%). For Klebsiella pneumoniae, low resistance was seen for tigecycline (0%) and minocycline (16%). Overall, ICU mortality was 31.3%, including miscellaneous cases.

Conclusion: Gram-negative infections, especially by multidrug-resistant organisms, are on the rise in ICUs. Empirical antibiotics should be used according to the local unit specific data. Constant evaluation of current practice on basis of trends in multidrug resistance and antibiotic consumption patterns are essential.

## Introduction

Sepsis and septic shock are the leading causes of mortality and morbidity in critical care units. After obtaining samples for cultures, antibiotics are started empirically in intensive care units (ICUs) to cover common pathogens causing sepsis. Early provision of adequate antibiotics improves survival outcomes among critically ill patients with infection. The early use of antibiotics provides effective control of infections; however, at the same time, the use of broad-spectrum empiric antibiotics is causing an increasing emergence of antibiotic resistance [1]. A rise in multidrug-resistant bacteria is limiting the available therapeutic options for infections in the ICU and further reducing the likelihood that empiric treatment selections will offer adequate coverage for common ICU pathogens [2].

Over the past two decades, there has been a rapid emergence of multidrug-resistant organisms, which is a major problem in terms of infection control [3]. Although 5% to 10% of all patients are treated in intensive care units (ICUs), they constitute about 25% of all nosocomial infections. The incidence is five to 10 times higher than in general hospital wards [4], which means that nosocomial infections are especially prominent in the ICU [5].

There is a wide diversity between institutions in the prevalence of pathogens and in their antimicrobial susceptibility [6]. The hospital antibiogram is a periodic summary of antimicrobial susceptibilities of local bacterial isolates. Antibiograms are often used by clinicians to assess local susceptibility rates as an aid in selecting an empiric antibiotic therapy and in monitoring resistance trends over time within an institution [7]. Therefore, this study aims to determine the types and frequency of microorganisms causing sepsis as well as to determine the antibiotic susceptibility and resistance of microorganisms isolated in medical ICU.

## Materials and methods

After approval from the institutional review board (IRB) at Shifa International Hospital, Islamabad Pakistan (approval #558-006-2016), a cross-sectional study was conducted in which data was collected from patients admitted to the medical intensive care unit over a one-year period from August 2015 to August 2016. Pediatric patients were excluded from the survey. Signed informed consent was obtained from every patient before enrollment in the study. Specimens collected were collected from blood, urine, endotracheal secretions, catheter tips, tissue, pus/pus swabs, cerebrospinal fluid (CSF), ascites, bronchoalveolar lavage (BAL), and pleural fluid (Table 1). All samples were inoculated and incubated for 24 to 48 hours. Colonies were subjected to gram staining and gram-negative and gram-positive organisms isolated.

**Table 1:** Source of Samples Taken and Organisms Cultured *cerebrospinal fluid (CSF); **bronchoalveolar lavage (BAL); Ɨ methicillin-resistant Staphylococcus aureus (MRSA); Ɨ Ɨ methicillin-sensitive Staphylococcus aureus (MSSA); ǂvancomycin-resistant enterococcus (VRE)

Organisms	Total Sample (n = 568)	Percentage (%)	Blood	Urine	Tracheal Secretions	Catheter Tip	Tissue	Pus/Pus Swab	CSF*	Ascites	BAL**	Pleural Fluid
Acinetobacter baumanii	87	15.3	11.6	0.6	28.0	29.7	0.0	4.2	0.0	33.3	18.2	0.0
Burkholderia cepacia	3	0.5	0.0	0.0	1.5	0.0	0.0	0.0	0.0	0.0	0.0	0.0
Candida albicans	47	8.3	4.5	25.3	0.0	2.7	0.0	4.2	0.0	0.0	0.0	0.0
Candida kefyr	1	0.2	0.0	0.6	0.0	0.0	0.0	0.0	0.0	0.0	0.0	0.0
Candida krusei	2	0.4	0.0	1.3	0.0	0.0	0.0	0.0	0.0	0.0	0.0	0.0
Candida parapsilosis	2	0.4	1.8	0.0	0.0	0.0	0.0	0.0	0.0	0.0	0.0	0.0
Candida rugosa	2	0.4	0.9	0.0	0.0	2.7	0.0	0.0	0.0	0.0	0.0	0.0
Candida spp.	27	4.8	0.0	17.1	0.0	0.0	0.0	0.0	0.0	0.0	0.0	0.0
Candida tropicalis	23	4.0	1.8	12.7	0.0	0.0	0.0	0.0	0.0	11.1	0.0	0.0
Enterobacter	13	2.3	2.7	0.6	2.5	2.7	0.0	8.3	0.0	0.0	9.1	0.0
Enterococcus	40	7.0	17.0	7.0	0.0	13.5	14.3	12.5	0.0	0.0	0.0	0.0
Escherichia coli	87	15.3	17.9	19.0	13.0	5.4	14.3	20.8	0.0	11.1	9.1	0.0
Klebsiella pneumoniae	58	10.2	9.8	5.7	13.5	16.2	14.3	0.0	0.0	22.2	0.0	100.0
Morganella morganii	2	0.4	0.0	0.6	0.0	0.0	7.1	0.0	0.0	0.0	0.0	0.0
Proteus mirabilis	5	0.9	0.0	0.6	1.0	2.7	7.1	0.0	0.0	0.0	0.0	0.0
Pseudomonas aeruginosa	74	13.0	10.7	5.7	19.0	13.5	7.1	8.3	50.0	11.1	45.5	0.0
Salmonella typhi	2	0.4	1.8	0.0	0.0	0.0	0.0	0.0	0.0	0.0	0.0	0.0
Serratia marcescens	5	0.9	0.9	0.0	0.5	0.0	7.1	8.3	0.0	0.0	0.0	0.0
MRSAƗ	35	6.2	4.5	0.0	11.0	0.0	14.3	12.5	0.0	11.1	18.2	0.0
MSSAƗƗ	23	4.0	5.4	0.0	6.5	0.0	7.1	12.5	0.0	0.0	0.0	0.0
Stenotrophomonas	6	1.1	2.7	0.0	1.0	2.7	0.0	0.0	0.0	0.0	0.0	0.0
Streptococcus spp.	4	0.7	1.8	0.0	0.0	0.0	0.0	8.3	0.0	0.0	0.0	0.0
Streptococcus pneumoniae	8	1.4	1.8	0.0	2.5	0.0	0.0	0.0	50.0	0.0	0.0	0.0
Trichosporon	1	0.2	0.0	0.6	0.0	0.0	0.0	0.0	0.0	0.0	0.0	0.0
VREǂ	11	1.9	2.7	2.5	0.0	8.1	7.1	0.0	0.0	0.0	0.0	0.0

Antibiotic susceptibility testing was done by the disk diffusion method according to the Clinical and Laboratory Standards Institute (CLSI) recommendations [8]. The antibiotics used were ampicillin, erythromycin, cloxacillin, amoxicillin/clavulanic acid, amikacin, ceftazidime, cefoperazone/sulbactam, piperacillin/tazobactam, ceftriaxone, imipenem, meropenem, ciprofloxacin, gentamicin, doxycycline, penicillin, colistin, co-trimoxazole, moxifloxacin, chloramphenicol, nitrofurantoin, fosfomycin, minocycline, clindamycin, vancomycin, and linezolid. The zone of inhibition was measured and interpreted as susceptible, intermediate, or resistant. Binary logistic regression analysis and Chi-square test (*X^2^*) were done to establish a relationship between the length of ICU stay and mortality.

## Results

**Figure 1:**
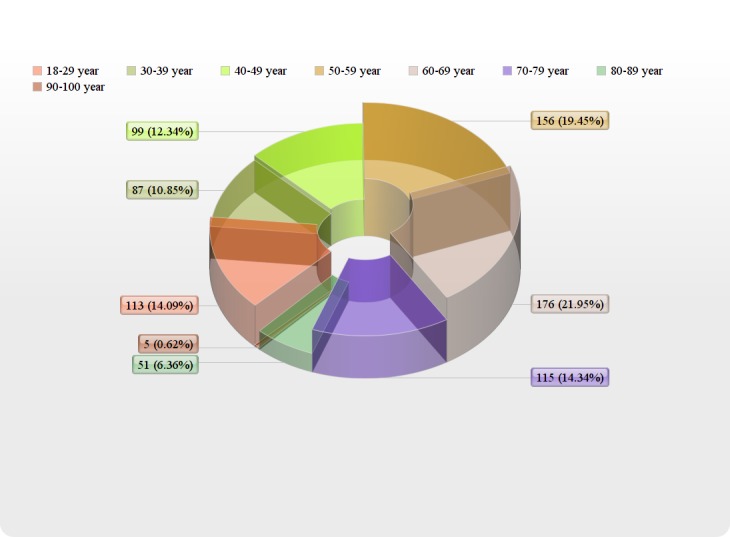
Age Distribution of Patients Admitted in the Intensive Care Unit

Of the total of 802 patients, 454/802 (56.6%) were males and 348/802 (43.3%) were females. Age distribution of patients admitted to the ICU is shown in Figure 1.

**Figure 2:**
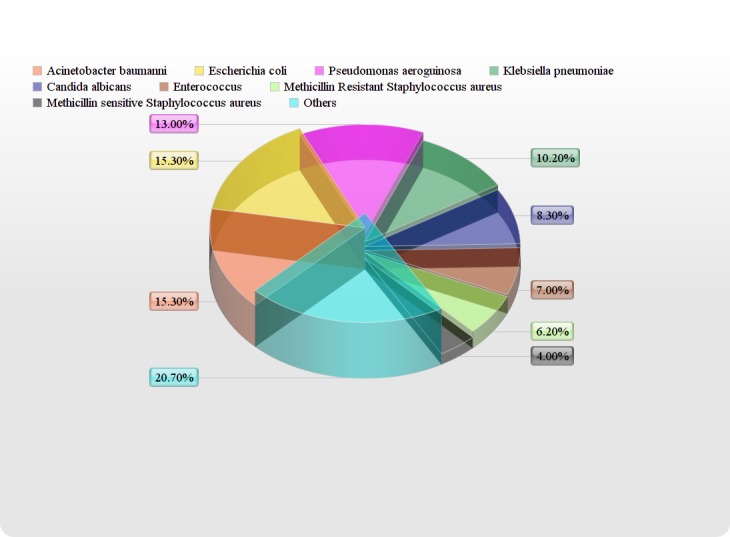
Organisms Cultured in Intensive Care Unit Patients

Three hundred twenty-eight patients (40.89%) showed positive cultures out of which 265/328 (81.5%) were bacterial isolates and 64/328 (18.5%) were Candida albicans. The most frequent isolated gram-negative bacteria were Acinetobacter baumannii (15.3%), Escherichia coli (15.3%), Pseudomonas aeruginosa (13%), and Klebsiella pneumoniae (10.2%). Other less frequent gram-negative bacteria included Enterobacter (2.3%), Stenotrophomonas (1.1%), Proteus mirabilis (0.9%), Serratia marcescens (0.9%), Burkholderia (0.5%), Morganella morganii (0.4%), and Salmonella typhi (0.4%) (Figure 2).

**Figure 3:**
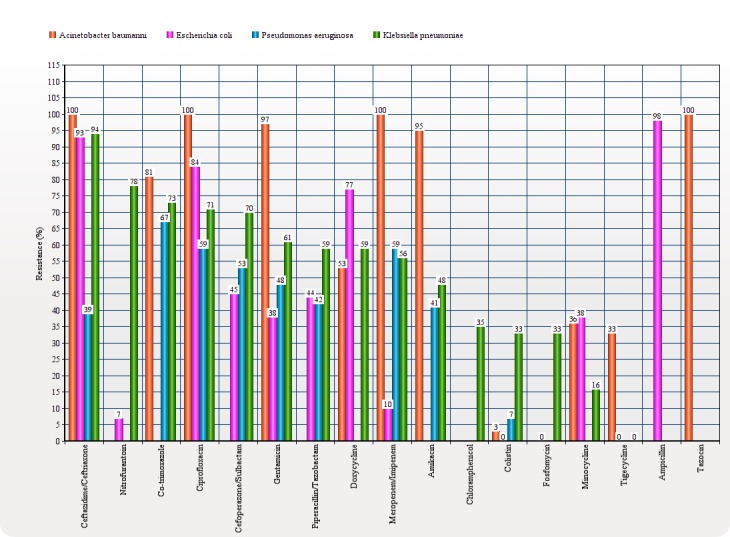
Spectrum of Antibiotic Resistance to Bacteria

Gram-positive isolates were less frequent as compared to gram-negative isolates and included Enterococcus (7%), methicillin-resistant Staphylococcus aureus (MRSA) (6.2%), methicillin-sensitive Staphylococcus aureus (MSSA) (4%), vancomycin-resistant enterococcus (VRE) (1.9%), Streptococcus pneumonia (1.4%), and Streptococcus spp (0.7%). In gram-negative isolates, for Acinetobacter, colistin was the most effective antibiotic (3% resistance), followed by tigecycline (33% resistance) and minocycline (36% resistance). For E.coli, colistin (0%), tigecycline (0%), amikacin (7%), and carbapenems (10% for both imipenem and meropenem) showed low resistance whereas nitrofurantoin and fosfomycin showed more sensitivity for E.coli urinary tract infections. For Pseudomonas aeruginosa, low resistance was seen for colistin (7%), ceftazidime (39%), and amikacin (41%). For Klebsiella pneumoniae, low resistance was seen for tigecycline (0%), minocycline (16%), and colistin (33%). In gram-positive isolates, for Enterococcus, vancomycin (0% resistance), linezolid (0% resistance), and chloramphenicol (15% resistance) were the most effective antibiotics, whereas, for MRSA, vancomycin, linezolid, doxycycline, and chloramphenicol showed 0% resistance and co-trimoxazole showed 17% resistance (Figure 3).

The length of ICU stay has a negative linear relationship with final outcome in terms of either transferring outpatient with stable vitals or mortality (r = -0.129, *X^2^*: p = 0.0001) (Table 2).

**Table 2:** Cross-Tabulation Analysis of Outcome and Length of ICU stay

Outcome	Length of ICU Stay						Total	Pearson Correlation Coefficient (r)	*X2: *p-value
	1-3 days	4-6 days	7-9 days	10-12 days	13-15 days	≥ 16 days			
Transferred out (n)	286	159	57	26	9	14	551	-0.129	0.0001
Expired (n)	180	43	12	7	4	5	251		
Total	466	202	69	33	13	19	802		

## Discussion

The use of antibiograms to help select empirical antibiotic therapy for suspected infection with likely or known pathogens is a well-established practice. In our study, out of the total bacterial isolates, gram-negative bacteria were more prevalent than were gram-positive bacteria. This predominance of gram-negative bacteria is in concordance with the findings of the similar study conducted in 2012 (Al-Jawady, et al.), which may be due to their wide prevalence in the hospital environment. In addition, their frequent resistance to antibiotics may play a role in their persistence and spread [9].

The predominant gram-negative isolates in our study were Acinetobacter and E. coli, whereas the study done by Rajan and Rao showed Klebsiella as the most predominant organism [10]. In a similar study, Ziab, et al. [11] reported Pseudomonas aeruginosa as the predominant gram-negative bacilli being isolated from the ICU, whereas E. coli was the most common gram-negative bacillus reported in studies done by Al-Jawady, et al. and Morfin-Otero, et al. [9, 12]. The most frequent isolate from urine was found to be E. coli in our study. This is comparable to studies conducted by Rajan, et al., Kritupanta, et al., and Sankarankutty, et al. [10, 13-14]. Acinetobacter was most predominant isolate from the respiratory tract in our study. A similar study conducted by Pradhan, et al. showed Acinetobacter to be the most frequent microorganism in respiratory tract [15]. Klebsiella was the most reported organism isolated in the respiratory tract in studies done by Rajan, et al. and Patel, et al. [10, 16].

Our study shows a very high prevalence of carbapenem resistance among Acinetobacter (100%). A similar study conducted by Mumtaz, et al. [17] has reported 79% resistance to imipenem, while Rajan, et al. [10] showed 52% carbapenem resistance among Acinetobacter. In our study, Acinetobacter was highly resistant to third generation cephalosporins (100% ceftazidime), aminoglycosides (97% gentamicin and 95% amikacin), and fluoroquinolones (100% ciprofloxacin and moxifloxacin). The most effective drug was colistin, which showed 3% resistance in our study. Similar results of colistin effectiveness against Acinetobacter were seen in the study by Rajan, et al. [10], while work published by Hasan, et al. [18] showed that tigecycline was the most effective antibiotic against Acinetobacter. Our study showed 33% tigecycline resistance to this bacterium. E. coli, in our study, showed high resistance to third generation cephalosporins (93% ceftazidime and 90% ceftriaxone); similarly, more than 90% E. coli were found to be resistant to third generation cephalosporin by Mohammadi-Mehr, et al. [19]. Carbapenem resistance was as low as 10% in our study. Almost similar results reported by Aysen, et al. [20] showed 13.1% E. coli resistance to imipenem. Gunjal, et al. [21] reported 28.10% of E. coli isolates were resistant to amikacin and 48.20% resistance to gentamicin, whereas we found 7% and 38% resistance to amikacin and gentamicin, respectively. Fosfomycin, colistin, and tigecycline showed no resistance in E. coli strains.

In our study, Pseudomonas showed significant resistance to carbapenems (59% imipenem/meropenem), whereas a study published by Rakhee, et al. [22] showed 20.8% resistance to imipenem and a study published by Rajan, et al. [10] showed 12.9% carbapenem resistance to Pseudomonas. Pseudomonas also showed high resistance to third generation cephalosporins (53% cefoperazone/sulbactam and 39% to ceftazidime) and aminoglycosides (48% gentamicin and 41% amikacin) in our study. Radji, et al. showed 60.9% resistance to ceftriaxone and found that amikacin was the most effective antibiotic against Pseudomonas with 15.6% resistance [23]. We found colistin to be the most effective antibiotic against Pseudomonas with only 7% resistance.

Klebsiella, the fourth most common microorganism of our study, showed high carbapenem resistance (56% meropenem and 55% imipenem), whereas Sheth, et al. [24] showed 100% sensitivity to carbapenems and Rajan, et al. [10] documented 28.13% carbapenem resistance. In our study, a high pattern of resistance was seen with third generation cephalosporins (94% ceftazidime, 82% ceftriaxone, and 70% cefoperazone/sulbactam) and aminoglycosides (61% gentamicin, 48% amikacin). Gunjal, et al. have reported 60% resistance to amikacin and 80% resistance to gentamicin [21]. Colistin was found to be the most effective antibiotic for multidrug-resistant (MDR) Acinetobacter, E. coli, and Pseudomonas, whereas tigecycline was found to be the effective antibiotic against multidrug-resistant Klebsiella.

### Limitations

External validation of this cross-sectional survey cannot be determined. The analysis is based on single centre experience and associated comorbidities have not been taken into account. These comorbidities might be contributing towards Neyman’s (prevalence) bias. The confounding factors, including age and gender, might affect overall ICU mortality.

## Conclusions

Multidrug-resistant gram-negative infections are on the rise in ICUs and are one of the contributory factors in the increase in overall ICU morbidity and mortality. Resistance to important antibiotic groups, including quinolones, piperacillin-tazobactam, and carbapenems, has increased substantially over the past few years. It is suggested that empiric antibiotics should be used according to the local ICU unit antibiograms. A constant evaluation of current practices on the basis of trends in multidrug resistance and antibiotic consumption patterns is essential.
